# Supplementary material for: Patterns of Intron Gain and Loss in Fungi
Source: PLoS Biol. 2004 Nov 30;2(12):e422. doi: 10.1371/journal.pbio.0020422 (PMC532390; doi:10.1371/journal.pbio.0020422)
Supplement: Table S1 — Also available at http://genes.mit.edu/NielsenEtAl/. (4.3 MB ZIP). [file pbio.0020422.st001.zip › NielsenEtAl/html/203.html]

AN7402.1.NCU01517.1.MG01096.1.FG06278.1


```
 CLUSTAL W (1.82) Multiple Sequence Alignments - Introns Inserted


Sequence 1: MG01096.1	649 aa
Sequence 2: FG06278.1	579 aa
Sequence 3: NCU01517.1	626 aa
Sequence 4: AN7402.1	661 aa
Alignment Length: 684 aa
Number Identitical Residues: 208 aa
Alignment Score (without introns) 12527


MG01096.1 	-----MD~ISRVDHGTSTSNITTSVNPVMHAISSFVLLGTYAVQAVLGRPNSLARR----
NCU01517.1	-------~--------------------MHLVSSLLVVG-AAFQAVLGLPDPLHEKRHSD
FG06278.1 	-------~--------------------MYFVSTALLFGSFALQSVLGRPALQERQ----
AN7402.1  	MPTTILK2ITLFPLIDSIFSVQLSPVRIAMLTLSKVLPVLALSHAVAAAPQLSARAT---
          	 .::  .  :      :  .   :         : ::      ::* . *    .     

MG01096.1 	---ANVDSFIEVERPIALQGVLRNIGSDGALVRGAGPG~VVIASPSTVDPDY~FYTWTRD
NCU01517.1	IIKRSVDSYIQTETPIAQKNLLCNIGASGCRASGAASG~VVVASPSKSSPDY1WYTWTRD
FG06278.1 	---SSLDSFIKSESSVAIEQLLCNIGSDGCHSQGVASG~VVIASPDTDDPDY1FFTWTRD
AN7402.1  	---ASLNTWLSTEASFALDGILTNIGANGAYAKTAKAG1-----------AD~YYTWTRD
          	    .:::::. * ..* . :* ***:.*.    . .*               ::*****

MG01096.1 	SALVLK~CLTDIFANKYDAGLQSTITQYITTQAQVQRIDNPSGPLSDGSGLGEPKYNANL
NCU01517.1	AALVTK~LIVDEFTNDYNTTLQNTIQAYAAAQAKLQGVSNPSGSLSNGAGLGEPKFMVDL
FG06278.1 	AALVFN2-----------------------------------------------------
AN7402.1  	AALTVK~VLVDLFHNG-DLSLQTILEEYTNSQAYLQTVSNPSGGLASG-GLAEPKFYVDM
          	:**. :    .   .  .   ..       :.:  .  ...:.  :.. . ....   . 

MG01096.1 	SPYLGSWG~RPQRDGPALRATALVAYANWLI~GNNYTSTARDIVWPILRNDLNYVAQYW2
NCU01517.1	QQFTGAWG~RPQRDGPPLRAIALIGYGKWLV~SNGYADTAKSIIWPIVKNDLAYTAQYW~
FG06278.1 	-PFTGGWG1RPQRDGPALRATAIITYANWLI~DNGYTSTASDIVWPVVRNDLNYVAQYW2
AN7402.1  	TAFTGSWG~RPQRDGPALRATTLIGFGNWLI0DNGYSSYASNNIWPIVRNDLTYVAQYW~
          	  : *.** *******.*** ::: :.:**: .*.*:. * . :**:::*** *.**** 

MG01096.1 	NRTGFD~LWEEVQGSSFFTTGAQYKA1LIEGDALAKKLGATCSSCESIAPNILCFLQSFW
NCU01517.1	NNTGFD~LWEEVNSSSFFTIAASHRA~LVEGSAFAKSVGSSCSACDAIAPQILCFQQSFW
FG06278.1 	NQTGFD~LWEEVKGSSFFTTASQYRA1LTEGAALAKKLGKPGDNYAAIAPQALCFLQTYW
AN7402.1  	SKSGYD1LWEEVNSMSFFTVAVQHRA~LVEGSTFAHRVGASCPWCDSQAPQILCYMQNFW
          	..:*:* *****:. **** . .::* * ** ::*: :* .     : **: **: *.:*

MG01096.1 	TPNGNYIVSNT~NTGTNRSGKDANSILSSIHSFDPAAGCDELTFQPCSAKSLANHKAVVD
NCU01517.1	SNSG-YIIS--~NFVNYRSGKDINSVLTSIHNFDPAAGCDVNTFQPCSDRALANHKVVVD
FG06278.1 	IPSGKYVDSNI1NVNDGRTGKDANSILTSIHNFDPSLSCDAATFQPCSDKALANHKAVTD
AN7402.1  	--TGSYINA--~NTGGGRSGKDANTVLASIHTFDPDAACDDITFQPCSSRALANHKVYTD
          	  .*.*: :   *    *:*** *::*:***.***  .**  ****** ::*****. .*

MG01096.1 	SFR-FYPINSGIQVGRGLAVGRYSEDVYYGGNPWHLTTLAAAEQIYDAVYVWKKQGRLEV
NCU01517.1	SMR-FWGVNSGRTAGKAAAVGRYAEDVYYNGNPWYLATLAAAEQLYDAVYVWKKQGSITV
FG06278.1 	SFR-SWNINKGISQGSAVSVGRYIEDVYYNGNPWYLATLAAAEQLYDALYVWKQKGSITV
AN7402.1  	SFRSVYSLNTGIAQGVAVAAGRYPEDSYYNGNPWFLTTLAAAEQLYDAIYQWQKARSISI
          	*:*: : :*.*   * . :.*** ** **.****.*:*******:***:* *::   : :

MG01096.1 	TSASLPFFRDLSSSIAVGSYASDSTTFTQIIDAALVYADGFIEVVQRYAGANGSLDEQID
NCU01517.1	TSTSLAFFKDLVPSVSTGTYSSSSSTYTAIINAVTTYADGFVDIVAQYTPSDGSLAEQFD
FG06278.1 	SDVSLSFFKDLVSGISTGTYASDSATFKSITDAVSKYADGYVSIVQKYVGDNGALAEQFD
AN7402.1  	TSTSLAFFKDIYSSAAVGTYASGSSAFTAIIDAVKTYADGYVSIVKAHAMANGSLSEQFD
          	:..**.**:*: .. :.*:*:*.*:::. * :*.  ****::.:*  :.  :*:* **:*

MG01096.1 	KNNGSQRSARDLTWSYAAFLTATDRRAGVVPPSWFV--PASLPGSCSTGSQQGVYSAVTP
NCU01517.1	KDSGAPLSATHLTWSYASFLSAAARRAGIVPPSWGAASANSLPGSCSASTVAGSYATATA
FG06278.1 	KNNGRPLSATDLTWSYAAFLSAADRRAGIVPPSWASG-AAAVPQQCGTQTVAGSYSLATA
AN7402.1  	KTYGTCVSARDLTWSYAALLTASMRRNGVVPPSWDAASANTLPSSCSTGSATGTYSTATV
          	*  *   ** .******::*:*: ** *:*****  .:. ::* .*.: :  * *: .* 

MG01096.1 	ASFPASQTPITGAPP---VPPTT-------TAPPGGCTTAPVVAVTFNVLVTTTPGDTIK
NCU01517.1	TSFPANLTP----------ASTTVT-----PPTQTGCAADHEVLVTFNEKVTTSYGQTVK
FG06278.1 	TSFPPSQTPKGGAPSPTGTQPSTSP-----TASPTSCPIATSVAVTFVETVTTNFGETIK
AN7402.1  	TTWPSTLTSGSASATTTIMATSTATSSSTTTSTTTACTTPSTVAVTFNVIATTTYGENVY
          	:::*.. *.  .:...:   .:* .:::::...  .*.    * ***   .**. *:.: 

MG01096.1 	IVGDIEDLGKWNPQNGIALDANDYTASRPLWKKAITLKAGQVVQYKFINVASGGAVRWEA
NCU01517.1	VVGSIAALGNWAPASGVTLSAKQYSSSNPLWSTTIALPQGTSFKYKYVVVNSDGSVKWEN
FG06278.1 	IVGNIPALGNWDTSKAVALSASDYTSSNPVWKATISLTAGQDIQYKYINVKKDGSVTWEK
AN7402.1  	IVGSISQLGNWDTGSAVALSASKNTSSNNLWYVDINLPGGTAFEYKYIRKETDGSIVWES
          	:**.*  **:* . ..::*.*.. ::*. :*   * *  *  .:**::   ..*:: ** 

MG01096.1 	DPNRVYTVPSSC-ATAVAVDGQWQ~T---------
NCU01517.1	DPDRSYAVGTDC-ASTATLDDTWR~----------
FG06278.1 	DPNRTYTVPKTC-ATKATKTDKWQ~S---------
AN7402.1  	DPNRSYTVPSSCGVSTATESDTWR2CTLETQSVRN
          	**:* *:* . *..: .:  . *:  : .:.:  .
```
